# Feeding difficulties during the neonatal period

**DOI:** 10.1186/1824-7288-41-S2-A21

**Published:** 2015-09-30

**Authors:** Luigi Corvaglia, Silvia Martini

**Affiliations:** 1 Neonatology and Neonatal Intensive Care Unit, S.Orsola-Malpighi Hospital, Department of Medical and Surgical Sciences, University of Bologna, Bologna, Italy

## 

Feeding difficulties (FD) are a major issue in neonatology, as they could hamper the assessment of an adequate enteral nutrition, delay hospital discharge and lead to breastfeeding failure.

Functional and anatomical maturation of the gastrointestinal tract is strictly related to gestational age (GA); hence, premature infants are more prone to develop FD. Feeding intolerance (FI) is very common among preterm infants; clinical symptoms of FI (e.g. abdominal distension, vomiting, bilious gastric residuals, occult or gross bloody stools) are observed in nearly 29% of such neonates [1]. FI could represent an early sign of necrotizing enterocolitis (NEC), which is the most feared gastrointestinal complication of prematurity. Hence, FI often brings clinicians to withhold, decrease or discontinue enteral feeds, thus hampering the establishment of an adequate enteral nutrition and leading to a prolonged duration of both parenteral nutrition (PN) and central lines, with increased risks of such complications as liver cholestasis or sepsis[2].

The coordination between sucking, swallowing and breathing is usually achieved at 34-36 weeks GA; hence, preterm infants are usually fed via an intragastric tube, through intermittent boluses or continuously. Poor sucking and sucking-swallowing incoordination are the major causes of FD and breastfeeding failure among late preterm infants (GA 34-36^6/7^ weeks), with an increased risk of hypoglycemia, excessive weight loss, hyperbilirubinemia, dehydration[3]. Due to FD, up to 27% of all late preterm infants need to be initially supplemented with intravenous fluids[4]; moreover, tube feeding is frequently required for feeding administration in the first days of life.

The abovementioned problems are infrequent in healthy term newborns. Term neonates developing FD such as poor sucking and/or vomiting need to be evaluated for pathological causes. Physical examination could aid to identify anatomical malformations possibly responsible for FD (e.g. cleft palate). FD and sleepiness can be due to hyperbilirubinemia, hypoglycemia or electrolyte disturbances[5], but could also subtend an underlying metabolic disease, such as hypothyroidism. FD, lethargy and/or other clinical neurological signs (e.g. seizures, focal neurological signs, hypo- or hypertonia, bulging fontanel, central apnoea) could address for central nervous system diseases (i.e. subarachnoid haemorrhage, ischaemic stroke, metabolic encephalopathy etc.). FD and lethargy could also represent a warning sign for invasive infections, especially if associated with respiratory distress, apnoea and bradycardia, temperature instability and increased capillary refill time[5]. Blood tests (including blood cells count, C-reactive protein, glucose, bilirubin, electrolytes, blood gas analysis) and cerebral ultrasound scan are useful tools to aid neonatologists in the differential diagnosis.
